# Supplementary material for: MetaRibo-Seq measures translation in microbiomes
Source: Nat Commun. 2020 Jun 29;11:3268. doi: 10.1038/s41467-020-17081-z (PMC7324362; doi:10.1038/s41467-020-17081-z)
Supplement: Supplementary file 10 — Supplementary Data 7 [file 41467_2020_17081_MOESM10_ESM.zip › File2/Confidence_High_Taxonomy/178231_out.krona.html]

Javascript must be enabled to view this page.

members
magnitude
magnitudeUnassigned
count
unassigned
taxon
rank

178231\_out

39

superkingdom
2
39

phylum
1239
39

39
186801
class

186802
order
39

39
541000
family

genus
946234
39

39

SRS011134\_contig\_number\_41336SRS013098\_contig\_number\_23207SRS013965\_contig\_number\_37930SRS014948\_contig\_number\_13550SRS014979\_contig\_number\_32769SRS016018\_contig\_number\_15854SRS016132\_contig\_number\_5609SRS018351\_contig\_number\_3213SRS018541\_contig\_number\_contig-100\_16285.16286SRS019496\_contig\_number\_1954SRS020328\_contig\_number\_5459SRS021219\_contig\_number\_7497SRS021948\_contig\_number\_26807SRS022609\_contig\_number\_5370SRS024009\_contig\_number\_9083SRS024132\_contig\_number\_contig-100\_2206.262849SRS056273\_contig\_number\_20755SRS063190\_contig\_number\_5150SRS065504\_contig\_number\_28533SRS074964\_contig\_number\_contig-100\_2072.2072SRS075078\_contig\_number\_28592SRS077294\_contig\_number\_contig-100\_39928.39928SRS078242\_contig\_number\_21145SRS078419\_contig\_number\_34726SRS097889\_contig\_number\_37182SRS097958\_contig\_number\_14267SRS098571\_contig\_number\_12296SRS098881\_contig\_number\_16680SRS1041031\_contig\_number\_contig-100\_20968.100792SRS1041140\_contig\_number\_20913SRS1054691\_contig\_number\_9601SRS1055095\_contig\_number\_contig-100\_378.379SRS143417\_contig\_number\_3641SRS143780\_contig\_number\_30204SRS144714\_contig\_number\_22130SRS146812\_contig\_number\_39560SRS148721\_contig\_number\_contig-100\_46.282218SRS149075\_contig\_number\_contig-100\_1429.42819SRS150029\_contig\_number\_contig-100\_3.125820
1193534
species
